# Evaluation of relative pollen productivities in temperate China for reliable pollen-based quantitative reconstructions of Holocene plant cover

**DOI:** 10.3389/fpls.2023.1240485

**Published:** 2023-11-07

**Authors:** Furong Li, Marie-José Gaillard, Siqi Xie, Kangyou Huang, Qiaoyu Cui, Ralph Fyfe, Laurent Marquer, Shinya Sugita

**Affiliations:** ^1^ School of Ecology, Sun Yat-Sen University, Shenzhen, China; ^2^ Department of Biology and Environmental Science, Linnaeus University, Kalmar, Sweden; ^3^ School of Earth Sciences and Engineering, Sun Yat-Sen University, Zhuhai, China; ^4^ Key Laboratory of Land Surface Pattern and Simulation, Institute of Geographic Sciences and Natural Resources Research, Chinese Academy of Sciences, Beijing, China; ^5^ School of Geography, Earth and Environmental Sciences, Plymouth University, Plymouth, United Kingdom; ^6^ Department of Botany, University of Innsbruck, Innsbruck, Austria; ^7^ Institute of Ecology, Tallinn University, Tallinn, Estonia

**Keywords:** China, relative pollen productivity, evaluation, observation of regional vegetation, REVEALS model

## Abstract

The Landscape Reconstruction Algorithm (LRA) is regarded as the soundest approach for quantifying taxon-specific plant cover from pollen data. The reliability of relative pollen productivity (RPP) estimates is fundamental in the accuracy of quantitative vegetation reconstruction using the LRA approach. Inconsistent RPP estimates produced by different studies can cast doubt on the reliability and applicability of quantitative vegetation reconstruction. Therefore, it is crucial that the RPP estimates are evaluated before being applied for quantitative vegetation reconstruction. We have tested two alternative approaches, namely, a leave-one-out cross-validation (LOO) method and a splitting-by-subregion strategy, using surface pollen assemblages and the REVEALS model—the first step in the LRA—to evaluate the reliability of RPPs estimates of 10 target taxa obtained in the cultural landscape of Shandong. We compared the REVEALS estimates (RVs) with observations of regional vegetation abundance (OBVs) and pollen proportions (PPs). The RVs of all taxa are generally closer to OBVs than PPs, and the degree of similarity depends strongly on the abundance of individual taxa in plant and pollen; taxa dominant in the region show the highest similarity between RVs and OBVs, such as *Artemisia*, Poaceae, and *Humulus*. The RVs of all herb taxa except *Humulus* and Asteraceae SF Cichorioideae are slightly overrepresented, and the RVs of all tree taxa are underrepresented except for *Castanea*. The comparison of RVs with OBVs collected from different spatial extents shows that the RVs of all herb taxa are more similar to OBVs collected from shorter distances (100 km and 75 km for the entire region and the subregion, respectively), whereas the RVs of all tree taxa are more similar to OBVs collected from longer distances (150 km and 100 km for the entire region and the subregion, respectively). Furthermore, our findings highlight the importance to test different sizes of area for vegetation surveys for evaluation of the RVs given that the appropriate size of vegetation survey may vary between low pollen producers (mainly herbs) and high pollen producers (mainly trees). We consider that the LOO strategy is the best approach in this case study for evaluating the RPP estimates from surface moss polsters. The evaluation confirms the reliability of the obtained RPP estimates for their potential application in quantitative reconstruction of vegetation abundance in temperate China.

## 1 Introduction

As an important part of the earth system, land cover plays an important role in the exchange of mass and energy of the climate system. It is therefore of great importance to incorporate land-cover information in climate modelling (e.g. Gaillard et al., 2010). Pollen records have been used to infer long-term vegetation changes for a century since von Post (1916) presented the potential of pollen assemblages for representing the surrounding vegetation. However, quantitative reconstructions of past vegetation across space and over time require a better understanding of factors that affect the pollen representation of surrounding vegetation. The two most notable factors that bias the representation of vegetation in pollen assemblages are the differences in the pollen production of different plant species and the dispersal ability of different pollen types. Taxa with high pollen production and efficient dispersal ability are usually overrepresented in pollen assemblages, whereas taxa with low pollen production and poor dispersal ability are underrepresented (e.g., Davis, 1963; Andersen, 1970; Parsons and Prentice, 1981). The correction of the representation bias is approached by modeling the relationships between pollen and surrounding vegetation (Davis, 1963; Andersen, 1970; Parsons and Prentice, 1981; Prentice and Parsons, 1983), and it is fundamental to vegetation reconstruction approaches. Among these approaches, the Extended R-value (ERV) models (Parsons and Prentice, 1981; Prentice and Parsons, 1983; Sugita, 1993) are now commonly used to infer taxon-specific pollen–vegetation relationships and generate relative pollen productivity (RPP) estimates from modern pollen and vegetation data. RPP estimates are necessary for running the Landscape Reconstruction Algorithm (LRA; Sugita, 2007a; Sugita, 2007b), which quantifies past plant abundance at regional and local scales, and the Multiple Scenario Approach (MSA; Bunting and Midleton, 2009), an approach that can test conceptual spatial arrangements and levels of taxon-specific plant cover in the past.

The LRA is a two-step modeling process to estimate past regional vegetation cover (using the REVEALS model: Sugita, 2007a) and local vegetation cover (using the LOVE model: Sugita, 2007b). The LRA incorporates the mechanism and factors within the relationship between modern pollen and vegetation into reconstructions of past vegetation. Furthermore, it reconstructs vegetation from different spatial scales by introducing the size of sediment basins into the model. The LRA has been evaluated using simulation approaches (Sugita, 2007a; Sugita, 2007b) and empirically in North America (Sugita et al., 2010) and many parts of Europe (e.g., Hellman et al., 2008a; Cui et al., 2014; Hjelle et al., 2015; Trondman et al., 2016; Marquer et al., 2020). The results show that the LRA works well in predicting the percentage cover of large vegetation units (e.g., Hellman et al., 2008a; Hellman et al., 2008b; Cui et al., 2014). In Europe, REVEALS-based maps (e.g., Pirzamanbein et al., 2014; Trondman et al., 2015; Githumbi et al., 2022) of vegetation composition and landscape openness were shown to be appropriate for climate modeling (Strandberg et al., 2014; Strandberg et al., 2022) and in evaluating the anthropogenic land-cover change scenarios (Kaplan et al., 2017) and dynamic vegetation models (Marquer et al., 2017; Dallmeyer et al., 2023). The LOVE model has been used to address biogeographical questions (e.g., Cui et al., 2013) and understand local-scale transformation of vegetation by people in archaeological research (e.g., Mehl and Hjelle, 2016; Fyfe et al., 2018).

Fundamental to the quantitative reconstruction of vegetation by using the REVEALS and LOVE models are reliable RPP estimates. Over recent years, a number of studies have been conducted to calculate RPP estimates using the ERV model worldwide, such as in North America (Calcote, 1995; Sugita, 1998), in Europe (latest review in Githumbi et al., 2022), and in China (e.g., Li et al., 2011; Wang and Herzschuh, 2011; Wu et al., 2013; Xu et al., 2014; Ge et al., 2015; Li et al., 2015; Li et al., 2017; Zhang et al., 2017; Jiang et al., 2020; Zhang et al., 2020; Wan et al., 2022). This effort has resulted in the application of the REVEALS model in studies in northern China (Wang and Herzschuh, 2011; Xu et al., 2014; Li et al., 2020; Li et al., 2023). Synthesis of RPP estimates in China and Europe found that the ranking of RPP estimates of major plant types is more or less consistent among studies. However, discrepancies exist between the estimated values from different studies (Broström et al., 2008; Mazier et al., 2012; Bunting et al., 2013; Li et al., 2018; Wieczorek and Herzschuh, 2020; Serge et al., 2023). RPP estimate validation is not common in China, with only single studies of key biogeographical zones: in temperate China (Xu et al., 2014), subtropical China (Jiang et al., 2020), and tropical China (Wan et al., 2022). Evaluation of the reliability of the available RPP estimates is crucial to having confidence in the accuracy of the REVEALS estimates, as inconsistent estimates of RPP values by different studies can cast doubt on the reliability and applicability of quantitative vegetation reconstruction (Hellman et al., 2008a; Hellman et al., 2008b; Soepboer et al., 2010; Trondman et al., 2015). Therefore, it is necessary to evaluate the RPP estimates that are being applied in the REVEALS model for quantitative vegetation reconstruction.

The reliability of RPP estimates can be evaluated by comparison of REVEALS estimated vegetation on pollen records from large lakes and observed vegetation around the lakes (Hellman et al., 2008a). However, in regions that only have a sparse distribution of large lakes (such as the mountain regions of China), the usage of this validation strategy is hampered. In theory, multiple small sites can be utilized to estimate regional vegetation composition (Sugita, 2007a), and this has been further tested with empirical data in southern Sweden (Trondman et al., 2016). The REVEALS estimates from multiple small bogs are comparable with that of relevant large lakes, although standard errors (SEs) of estimates from multiple small sites will be larger than the ones from large lakes due to between-site variation (Sugita, 2007a; Trondman et al., 2016). This approach has been used to validate the LRA in Denmark (Nielsen, 2004) and North America (Sugita et al., 2010). A second approach to evaluating relative pollen productivity estimates is via simulated pollen and vegetation data (Sugita, 2007a).

In this study, we aim to (1) rigorously evaluate the relative pollen productivity estimates originally obtained from the cultural landscape of Shandong (Li et al., 2017), with detailed vegetation data that cover a large enough spatial extent, taking advantage of the strength of the ERV and the REVEALS model, and with the possibility of quantifying uncertainty and (2) compare two alternative strategies for evaluating these RPP estimates and propose possible approaches for future validation of RPP estimates.

## 2 Materials and methods

### 2.1 Geographical setting and site characteristics

**Figure 1:**
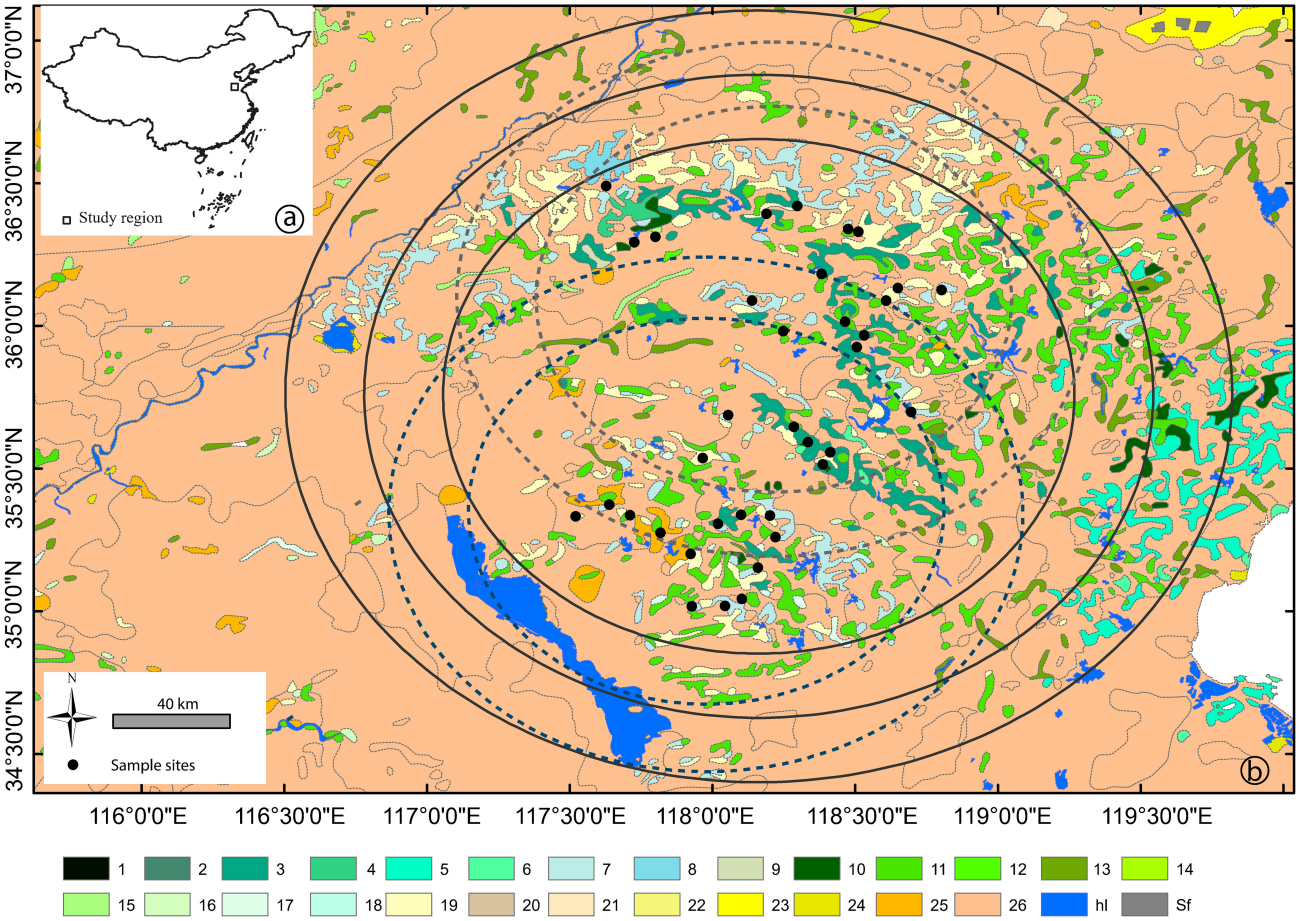
**(A)** Location of the study area and **(B)** distribution of the sampling sites with related areas for vegetation data of different size ("buffers"); blue (SW sites) and grey (NE sites) dashed circles indicate distances (buffers) of 75 km and 100 km in strategy I, and black solid circles indicate distances (buffers) of 100 km, 125 km, and 150 km in strategy II (see **Figure 2** for details on validation strategies). Vegetation communities: 1. *Picea jezoensis* forest; 2. *Juniperus komarovii* forest; 3. *Pinus tabuliformis* forest; 4. *Pinus tabuliformis and Robinia pseudoacacia* mixed forest; *5. Pinus densiflora* forest; 6. *Pinus thunbergii* forest; 7. *Platycladus orientalis* forest; 8. *Platycladus orientalis* and *Bothriochloa ischaemum* steppe; 9. *Platycladus orientalis* and *Quercus variabilis* mixed forest; 10. *Quercus acutissima* forest; 11. *Robinia pseudoacacia* forest; 12. *Malus sieversii* forest; 13. *Salix matsudana* forest; 14. *Populus simonii* forest; 15. *Populus nigra* forest; 16. *Populus*, *Salix*, *Ulmus* mixed forest; 17. *Cotinus coggygria* shrub; 18. *Carex moorcroftii* steppe; 19. *Vitex negundo*, *Ziziphus jujuba*, *Bothriochloa ischaemum* shrub–steppe; 20. *Zoysia japonica*-dominated meadow; 21. *Secale sylvestre* and *Leymus paboanus*-dominated meadow with *Betula pendula*; 22. *Aeluropus pungens*-dominated meadow; 23. *Suaeda glauca*-dominated meadow; 24. *Phragmites australis* Marsh; 25. cultivated crops: *Triticum aestivum*, *Oryza sativa*, *Setaria italica*, *Ipomoea batatas*, *Sorghum bicolor*, *Gossypium* spp., *Arachis hypogaea*, *Zea mays*; 26. cultivated crops with cultivated trees (*Juglans regia*, *Castanea mollissima*, *Crataegus pinnatifida*, *Malus domestica*, *Amygdalus persica*, *Prunus armeniaca*, *Vitis vinifera*, *Ziziphus jujuba* var. jujuba); *hl.* waterbody; *Sf.* saltern.

The study region, located in the lower reach of the Yellow River drainage basin (**Figure 1**) is one of the most important agricultural regions of China. The potential natural vegetation is deciduous mixed forest according to Wu (1980). At present, the region is characterized by cultivated modern fields in the plains, and traditional land use on the terraces in the low mountain areas. The study region encompasses 16 land-cover categories grouped into five broader vegetation communities: (i) cultivated crops dominated by *Triticum* spp. (wheat), *Zea mays* (maize), *Arachis hypogaea* (peanut), and *Ipomoea batatas* (sweet potato); (ii) cultivated fruit and nut trees consisting of *Castanea mollissima* (chestnut), *Crataegus pinnatifida* (Chinese hawthorn), *Diospyros kaki* (kaki), *Juglans regia* (walnut), *Malus domestica* (apple), *Pyrus* spp. (pear), *Prunus cerasus* (cherry), and *Zanthoxylum bungeanum* (Chinese pepper); (iii) secondary woodland including mainly *Pinus tabuliformis* (Chinese red pine), *P. thunbergii* (black pine), *Platycladus orientalis* (Chinese thuja), and *Robinia pseudoacacia* (black locust); (iv) ruderal community characteristic of abandoned or managed agricultural area boundaries; and (v) meadows dominated by *Artemisia mongolica* (Besser), *A. annua* (sagewort), Asteraceae, Caryophyllaceae, *Humulus scandens* (hop), *Lespedeza bicolor* and *L. tomentosa* (bush clovers), and *Vitex negundo* (Chinese chaste tree). More detailed information on the spatial distribution of the vegetation in this region is presented in Li et al. (2017).

### 2.2 Modern pollen data collection

A total of 36 surface moss polsters were collected randomly within an area of 100-km radius in May 2013 and 2014, among which 17 and 19 sites are located within an area of around 50-km radius in the northeast and southwest, respectively. The sample collection and pollen extraction strategy were described in Li et al. (2017). Pollen identification was performed under a light microscope at 400× magnification, and in special cases at 600× magnification, by using the pollen flora of China (Wang, 1995) and the European pollen flora (Beug, 2004) as a complimentary, and reference collections of physical specimens at the Paleoecology laboratory (Linnaeus University, Sweden) Pollen counts were limited to no less than 1,000 grains produced by terrestrial flowering plants for each sample.

### 2.3 Vegetation data surrounding the sampling sites

For evaluation of the RPP estimates obtained by Li et al. (2017), we collected the observed local- and regional-scale plant cover for the region. We operationally define the local and regional scale as within 1,500 m from each site and up to 150 km from the geographical center of all sites, respectively.

#### 2.3.1 Observed local vegetation survey within 1,500 m of the sampling sites

Vegetation surrounding the 36 moss-polster sites was surveyed up to 1,500 m for each site following the standard protocol suggested by Bunting et al. (2013) during the same period as pollen sample collecting, where the vegetation survey strategy varies depending on the distance from the sampling sites (zones A, B, and C) and is briefly summarized as follows. (1) Zone A (0−10 m): plant cover was estimated within 21 × 1 m^2^ quadrats, one quadrat was located at the center of the sampling site, and 20 other quadrats were placed at specific distances of N, E, S, W, NE, SE, SW, and NW directions. (2) Zone B (10−100 m): vegetation communities, defined based on dominant taxa, were mapped using a compass and a handheld GPS out to 100 m, and vegetation was surveyed in each community within several randomly distributed 1-m^2^ quadrats for the open community, 6-m radius point surveys and 1-m^2^ quadrats for semi-open and forest community. (3) Zone C (100 m–1,500 m): Google Earth image within 1,500 m from the center of each moss sample was used to identify land-cover categories, where both winter and summer images were adopted to separate deciduous from evergreen trees; plant composition for each land-cover category was obtained from the field surveys. For a more detailed vegetation survey strategy, the readers are referred to Li et al. (2017).

#### 2.3.2 Observed regional plant cover

To compare the REVEALS estimates with observed vegetation within different distances (or buffers), we obtained the plant cover within a radius of 100 km, 125 km, and 150 km from the geographical center of the 36 sampling sites. Meanwhile, the mean plant cover for the two subregions was obtained within a circle of 75-km and 100-km radius from the geographical center of the 17 sites and 19 sites, respectively (**Figure 1**). The distances were selected based on previous empirical and theoretical studies, which show that REVEALS estimates represent vegetation within an area of ca. 10^4^–10^5^ km^2^ (e.g., Soepboer et al., 2007; Sugita, 2007a; Hellman et al., 2008a; Wan et al., 2022).

The extracted regional plant abundance was compiled from three data sources. (1) Land-cover types were primarily extracted from a 1:1 million vegetation map of China (Hou, 2019); this data source provides 542 Chinese land-cover categories, of which 23 were included within the designed areal extent of this study. Plant composition for each categorized land-cover type was obtained from (2) a field survey as in Li et al. (2017), and (3) literature (Wang and Zhou, 2000). The field survey results were generalized by calculating the mean species abundance of all representative quadrats of the same categorized land-cover type. For land-cover categories whose plant composition is not available from the field survey, the information was obtained from the literature (Wang and Zhou, 2000). When the species composition in the literature is described as Braun-Blanquet cover-abundance scale (Braun-Blanquet, 1964), the abundances were translated to cover estimates within each vegetation type as follows: present—0.01%; I—5%; II—10%; III—25%; IV—50%; V—75%.

The plant species recorded in the field survey and collected from the literature were harmonized with pollen morphological taxonomy and nomenclature to overcome the variation in taxonomic resolution of pollen identification, following two publications (Wang, 1995; Beug, 2004). The most common ten pollen morphological types that were present in at least 60% of the sites and with a maximum percentage value larger than 1% were selected for this study. The plant composition (aggregated to pollen morphological taxonomy) of each categorized community is presented in **Table 1**.

**Table 1:** List of vegetation categories (the same as in **Figure 1**) and the plant composition (aggregated to pollen morphological taxonomy) of each categorized community that are included within the buffer distance of 150 km.

Vege code	Land cover type	Art	Caryo	Cas	Che	Cich	Hum	Pin	Poa	Que	Ulm
3	*Pinus tabulaeformis* forest^1^	4.82	1.69	0	0	1.74	0	74.5	20.1	2.2	6.25
5	*Pinus densiflora* forest^1^	4.82	1.69	0	0	1.74	0	74.5	20.1	2.2	6.25
6	*Pinus thunbergii* forest^1^	4.82	1.69	0	0	1.74	0	74.5	20.1	2.2	6.25
7	*Platycladus orientalis* forest^1^	0.89	0	0	1.78	0.63	2.08	0	11.5	0	0.76
8	*Quercus acutissima* forest ^1^	3	3.01	0	0.04	0.1	0.4	6.72	16	73.9	0
9	*Robinia pseudoacacia* forest^1^	1.74	0.8	0	2.48	1.3	0.3	1.82	26.3	0	0.08
10	*Malus sieversii* forest^2^	0	0	0	0	0	0	0	21	0	0
11	*Salix matsudana* forest^2^	0	0	0	0	0.1	0	0	103	0	0
14	*Populus*, *Salix*, *Ulmus* forest^2^	0	0	0	0	0.1	0	0	103	0	36
15	*Cotinus coggygria* ^2^	2	0	0	0	0	0	0	4	0	0
16	*Carex moorcroftii* ^2^	2.35	4.71	0	0	0	0	0	7.1	0	0
17	*Vitex negundo* var. *heterophylla*, *Ziziphus jujuba* var. *spinosa*, *Bothriochloa ischaemum* scrub, and grass^1^	1.8	4.41	0.69	0.17	0.63	0.15	0.04	20.1	0.07	1.85
22	*Phragmites communis* swamp^2^	0	0	0	0	0	0	0	92	0	0
23	Cultivated field^1^	0.35	0.05	0.97	0.6	1.86	0.15	0	1.05	0	0.01
24	Cultivated tree^1^	0.83	0.43	9.70	1.90	2.49	0.63	0.00	9.64	0	0.01

Plant composition for vegetation categories is collected from a field survey (Li et al., 2017; marked with^1^) and published literature (Wang and Zhou, 2000; denoted with^2^). Plant species harmonized to pollen-type full names: Art, *Artemisia*; Caryo, Caryophyllaceae; Cas, *Castanea*; Che, Amaranth/Chenop; Cich, *Asteraceae SF.* Cichorioideae; Hum, Cannabis/*Humulus*; Pin, *Pinus*; Poa, Poaceae; Que, *Quercus*; Ulm, *Ulmus*.

### 2.4 RPP estimate validation using the REVEALS model

**Figure 2:**
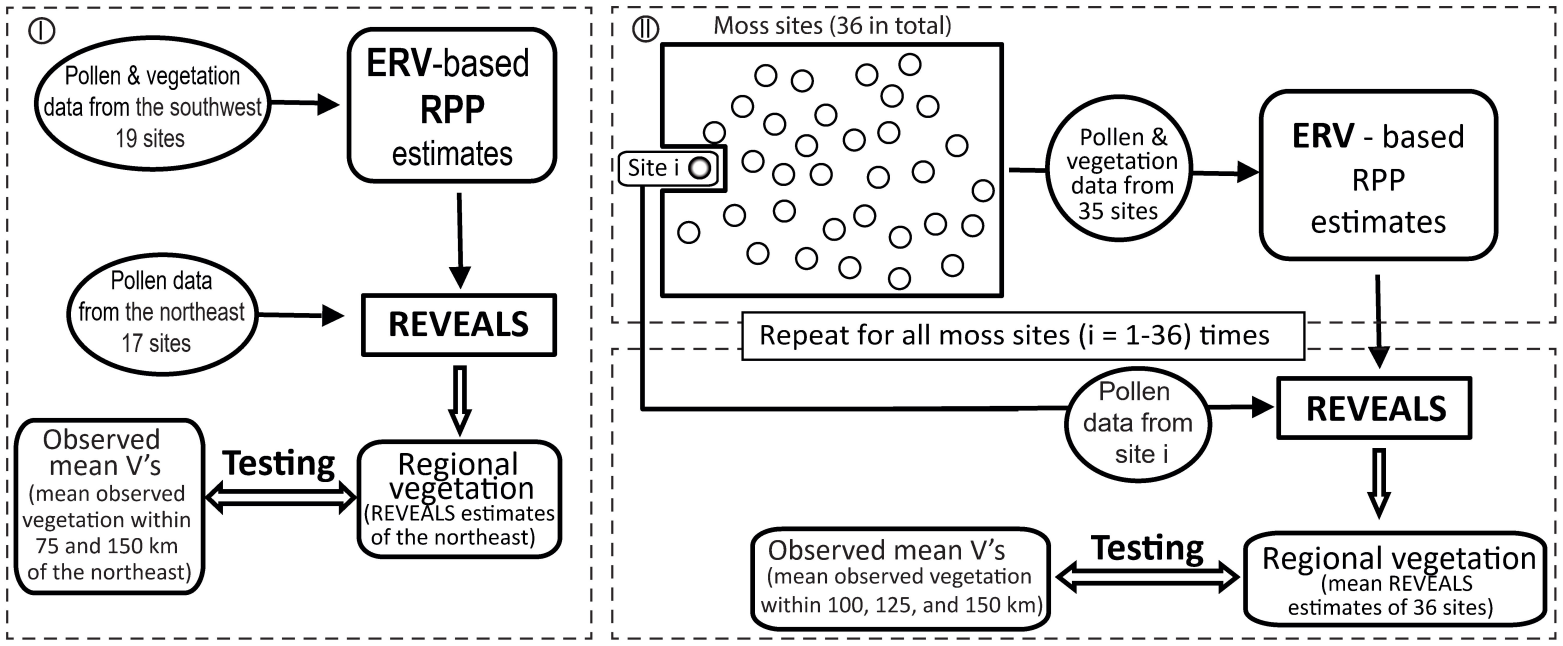
Flowchart of strategies used to validate relative pollen productivity (RPP) using the REVEALS model: the splitting-by-subregion strategy (I) and the leave-one-out (LOO) strategy (II).

To evaluate the relative pollen productivity (RPP) estimates from Shandong (Li et al., 2017), we use pollen counts from 36 moss polsters, observed local vegetation, and regional vegetation. When subfossil pollen data are available only from small-sized sites, such as within this region, REVEALS results have been shown to be robust when pollen data from several small-sized lakes and bogs are used, both theoretically (Sugita, 2007a) and empirically (Sugita et al., 2010; Fyfe et al., 2013; Trondman et al., 2016). In attempting to maximize the effectiveness of our validation, we have used two independent strategies to avoid circularity in the validation process for obtaining RPP estimates (we rerun the ERV model based on 10 target taxa of this study instead of the exact values based on 18 taxa published in Li et al. (2017)) and the REVEALS estimates. **Figure 2** summarizes the testing schemes.

In strategy I, we handle the pollen and vegetation data from the northeast and the southwest subgroups separately. We run the ERV model with pollen counts and vegetation data within 1.5 km of the 19 sites located in the southwest and the 17 sites located in the northeast of the study region to generate a new set of RPP estimates. The standard deviations (SDs) for all RPP estimates are lower than RPP values in the southwest, whereas the SDs of several taxa are larger than the RPP values in the northeast (results not shown), which motivated us to apply the REVEALS model on pollen counts from the 17 sites in the northeast and the RPP estimates obtained from the southwest. The 17 northeast moss polster samples are grouped within an area of around 50 km radius; we therefore compared the REVEALS estimates from the northeast with the observed regional vegetation from two different distances (75 km and 100 km) from the geographical center of the northeast to test the spatial scale of the REVEALS model. We refer to original pollen proportions as PPs, the REVEALS estimates of regional vegetation as RVs_NE, and the observed vegetation as OBVs_NE75 and OBVs_NE100, for distances of 75 km and 100 km respectively.

In strategy II, we use the “leave-one-out” (LOO) cross-validation scheme (Efron and Tibshirani, 1993; Sugita et al., 2010; Mazier et al., 2015). We remove one site from the full dataset of 36 locations and apply the ERV model using pollen and vegetation data from the remaining 35 sites to obtain RPP estimates. We then apply the REVEALS model using the new RPP values and pollen counts of the selected site that has been removed from the dataset (**Figure 2**). The process is repeated 36 times, and mean REVEALS estimates and related SEs from the 36 runs are calculated, where the SEs of the mean REVEALS estimates were calculated based on the delta method (Stuart and Ord, 1994). We refer to the mean REVEALS estimates of the regional vegetation obtained LOO as RVs_LOO, whereas the observed regional vegetation as OBVs_LOO100, OBVs_LOO125, and OBVs_LOO150 for distances of 100 km, 125 km, and 150 km respectively.

For all ERV model runs, Poaceae is chosen as the reference taxon for which the RPP is set to 1, and RPP of all other taxa is expressed relative to Poaceae. The fall speed of pollen (FSP), derived from Li et al. (2017), was originally calculated from Stoke’s law (Gregory, 1973) with measurements of the size along the short and long axes of the pollen grains. The taxon-specific approach of Prentice (Prentice, 1985) was selected to calculate distance-weighted plant abundance at a distance from the edge of the depositional basin (0.5-m radius), based on Sutton’s atmospheric diffusion model (Gaussian plume diffusion model, GPM) for the deposition of small particles in the air (Sutton, 1953). ERV (Extended R-value) submodel 3 (Sugita, 1994) was applied for the estimation of the RPP of each taxon with a maximum likelihood method (Parsons and Prentice, 1981; Prentice and Parsons, 1983; Sugita, 1994); the moving-window linear regression method (Gaillard et al., 2008) was used for estimating the relevant source area of pollen (RSAP) simultaneously. For a detailed rationale for parameter setting in the model run, the readers are referred to Li et al. (2017).

Except for the two strategies shown and mentioned above, we attempted to randomly select half the number of sites to run the ERV model, and the other half to run the REVEALS model, and only two among 10 trials showed promising results (results not shown). Others either have large SDs of RPP estimates from the ERV model or have too large SEs of the REVEALS estimates, hence they are not shown here. Moreover, we have attempted the strategies adopted by Sugita et al. (2010), which is using a randomly selected half-number of sites to run the ERV model to get RPP estimates and applying LRA with the LOO cross-validation strategy on the other half of sites. However, the SEs of the REVEALS estimates or LOVE estimates are too large (results not shown).

## 3 Results

These results describe the differences in the RPP estimates and the validation efficiency between the two strategies (splitting-by-subregion strategy (I) and leave-one-out strategy (II)) and using all pollen samples from the entire study region. This is done separately for the two strategies.

### 3.1 Results from strategy I

#### 3.1.1 Relevant source area of pollen and relative pollen productivity estimates

Log-likelihood against distance curves (ESM **Figure S1**) in ERV models indicate the change in goodness of fit of the data to the model, and log-likelihood (LLD) increases with distance. The point at which the curve reaches an asymptote is defined as the relevant source area of pollen (RSAP), measured in meters (Sugita, 1994). The curve of LLD approached the asymptote at a distance of 154 m when the southwest sites were used for running the ERV model, whereas the RSAP was 145 m both in the entire region and when all sites were used for calculating RPP estimates of 18 taxa (Li et al., 2017).

**Figure 3:**
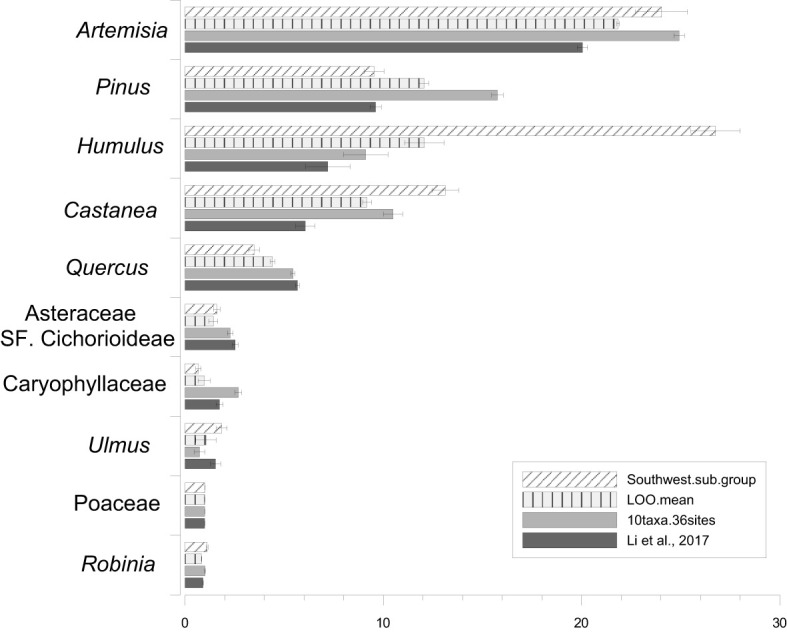
Bar plot of the relative pollen productivity (RPP) estimates and their standard deviations (SDs) of the 10 target taxa from the southwest, mean RPP estimates and related standard error of the LOO strategy, and the RPP estimates and SDs recalculated for the 10 taxa for the entire region and the RPP estimates from Li et al. (2017).

The RPP estimates from strategy I and the mean RPP values from strategy II are presented in **Table 2** and **Figure 3**, alongside recalculated RPP based on 10 target taxa from the entire region and earlier published RPP values (Li et al., 2017). The SDs for RPP estimates from the southwest and the entire region and the SE of mean RPPs from LOO are lower than RPP values. The ranking of the RPP estimates from the southwest is *Humulus* > *Artemisia* > *Castanea > Pinus > Quercus > Ulmus >* Asteraceae SF. Cichorioideae *> Robinia >* Poaceae *>* Caryophyllaceae. While the ranking is *Artemisia > Pinus > Humulus > Castanea > Quercus >* Asteraceae SF. Cichorioideae Caryophyllaceae*> Ulmus>* Poaceae*> Robinia* for the entire region.

**Table 2:** The relative pollen productivity (RPP) estimates from strategy I (the southwest subregion), the mean RPP estimates of strategy II (LOO) strategy, and recalculated RPP based on 10 target taxa from the entire region and earlier published RPP values (Li et al., 2017).

Taxa	Strategy I (subregion)	SD	Strategy II (LOO)	SE	Entire region (10 taxa, 36 sites)	SD	Li et al., 2017 (18 taxa, 36 sites)	SD
*Artemisia*	24.03	0.07	21.84	1.31	24.92	0.26	20.04	0.15
*Pinus*	9.54	0.24	12.05	0.51	15.76	0.29	9.61	0.23
*Humulus*	26.75	1.00	12.08	1.24	9.12	1.12	7.21	0.90
*Castanea*	13.13	0.23	9.18	0.67	10.50	0.48	6.07	0.34
*Quercus*	3.49	0.12	4.41	0.27	5.45	0.09	5.67	0.17
Asteraceae SF. Cichorioideae	1.60	0.23	1.43	0.16	2.28	0.15	2.53	0.11
Caryophyllaceae	0.68	0.31	0.97	0.14	2.69	0.17	1.74	0.16
*Ulmus*	1.85	0.51	1.06	0.27	0.73	0.27	1.55	0.37
Poaceae	1	0	1	0	1	0	1	0
*Robinia*	1.12	0.01	0.84	0.05	1.01	0.03	0.91	0.03

#### 3.1.2 REVEALS estimates

**Figure 4:**
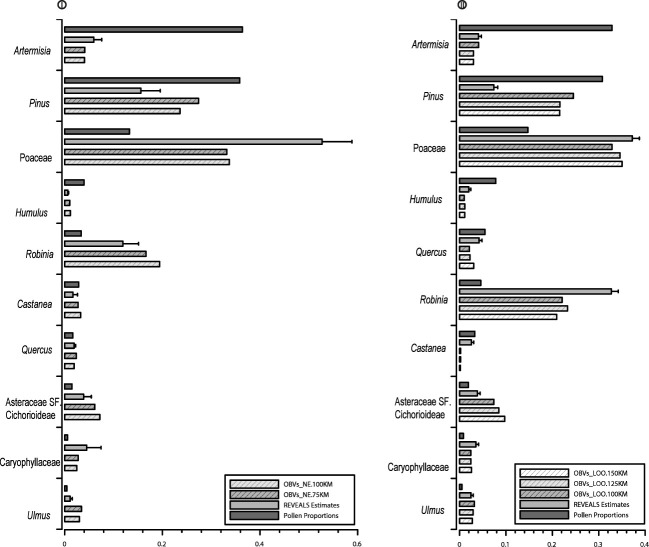
Bar charts of REVEALS-based vegetation abundance (RVs) in comparison with that of pollen proportions (PP) and observed vegetation (OBVs) within different distances from strategy I and strategy II.

To visualize the accuracy of the estimated vegetation using the REVEALS model, the RV_NE and PPs are shown in comparison with OBVs_ NE75 and OBVs_NE100 for each of the individual taxa (10 target taxa) from the strategy I (**Figure 4**). Poaceae, *Robinia*, Asteraceae SF. Cichorioideae, Caryophyllaceae, and *Ulmus* are underrepresented in PPs in comparison with OBVs. *Artemisia*, *Pinus*, *Humulus*, and *Castanea* are overestimated by PPs. The SEs for all RVs are lower than RV values, and the RVs of all taxa are generally closer to OBVs than PPs alone. RVs of all herb taxa, except *Humulus* and Asteraceae SF Cichorioideae, are slightly overrepresented, and RVs of all tree taxa are underrepresented. The comparison of RVs with OBVs collected at different spatial scales shows that the RVs of all herb taxa are closer to OBVs_NE75, whereas the RVs of all tree taxa (*Pinus*, *Robinia*, *Quercus*, *Castanea*, and *Ulmus*) are more similar to OBVs_NE100.

### 3.2 Results from strategy II

#### 3.2.1 Relevant source area of pollen and relative pollen productivity estimates

Log-likelihood values increased with distance, and the curves approached the asymptote at a distance between 150 and 171 m for all 36 ERV runs in the LOO strategy (ESM **Figure S1**), whereas the RSAP was 145 m based on the ERV using all samples from the entire study region and in Li et al. (2017).

The SDs for all RPP estimates from the 36 runs in strategy II are lower than RPP values, and the rank order of mean RPP estimates of the 36 ERV runs in the LOO strategy and the entire region is similar, which is *Artemisia* > *Humulus* > *Pinus* > *Castanea* > *Quercus* > Asteraceae SF. Cichorioideae > *Ulmus* > Poaceae > Caryophyllaceae > *Robinia*, except that *Pinus* has a higher RPP than *Humulus*, and Caryophyllaceae has a higher RPP than Poaceae and *Robinia* in the ERV runs of the entire region.

#### 3.2.2 REVEALS estimates

To visualize the accuracy of the estimated vegetation using the REVEALS model, **Figure 4** depicts the RVs and PPs, in comparison with OBVs from 100 km, 125 km, and 150 km for individual taxa from strategy II. Similar trends are displayed to those in strategy I: Poaceae, *Robinia*, Asteraceae SF. Cichorioideae, and Caryophyllaceae are underrepresented in PP, and *Artemisia*, *Pinus*, and *Quercus* are overestimated. SEs of RVs for all taxa are smaller than the RV values, and the REVEALS model improves the accuracy of estimated vegetation compared to PP alone for all taxa except *Castanea.* RV underestimates cover of *Pinus*, *Robinia*, and Asteraceae SF. Cichorioideae and slightly overestimates cover of *Artemisia*, Poaceae, Caryophyllaceae, and *Ulmus*. A comparison of the RVs with OBVs from different spatial scales show similar trends to strategy I. RVs are more similar to OBVs_LOO100 for *Artemisia* and Asteraceae SF. Cichorioideae, whereas RVs are more similar to OBVs_LOO125 for *Ulmus*, and closer to OBVs_LOO150 for *Pinus*, Poaceae, *Quercus*, and *Robinia.* There is no discernible difference between OBVs_LOO100, OBVs_LOO125, and OBVs_LOO150 km for *Humulus* and Caryophyllaceae.

## 4 Discussion

### 4.1 Relative pollen productivities

The stability/reliability of RPP estimates of the ERV runs is firstly evaluated by the shape of log-likelihood curve against distance. In all tests, the curve increased and reached an asymptote, which ensures the goodness of fit of the data to the model. However, the shape of the curve from strategy I (dividing the dataset by region) shows a slight departure from the theoretical behavior, which should be stable after having reached the asymptote. The ERV results from the LOO strategy and the whole data set are similar in terms of the changes in both the log-likelihood and the likelihood function score against distance (ESM **Figure S1**).

The stability/reliability of RPP estimates is highly correlated with their standard deviations (SDs) calculated in the ERV model. In this study, SDs of all RPP estimates from both strategies are lower than the RPP values. The ranking of the RPP estimates does not show significant differences between the southwest (strategy I), the LOO strategy (strategy II), and the entire region. The variation in RPP estimates between the three datasets is within the range of 30% for most of the taxa, except *Humulus*, Caryophyllaceae, and *Ulmus*, where the variation is up to 50% between estimates. Greater differences in RPP estimates are found between the southwest and the entire region than between the LOO strategy and the entire region.

Several factors may account for the dissimilarities between the RPP estimates. The noticeable disparity can be seen in the vegetation openness and heterogeneity caused by differences in human disturbance between the two subregions. There are more sites from the southwest subregion that are either located within cultivated fields or include cultivated fields within 100 m compared with the northeast. Nevertheless, there is little direct evidence to believe that differences in RPP estimates from the southwest and the entire region are due to human activity, although previous studies have found that pollen concentration of non-cultivated plants is much lower in cultivated fields than in the natural and abandoned field communities (e.g. Li et al., 2012). Therefore, it would be worth testing the impact of human activity on RPP estimates in the future.

### 4.2 Validation

#### 4.2.1 Evaluation of the validation strategy

There are various strategies to validate RPP estimates (Soepboer et al., 2007; Sugita et al., 2010; Jiang et al., 2020; Wan et al., 2022). In this work, we have evaluated RPPs in terms of the stability of RPP estimates, and similarities between observed and estimated regional vegetation based on the three sets of RPP estimates generated here.

We first compare the RPP estimates from the two strategies (LOO and regional splitting) with those based on 10 taxa of the entire region. The RPP estimates of all taxa from the LOO strategy, except for *Artemisia* and *Robinia*, are more similar to those of the entire region than those from the regional splitting approach (**Table 2**). The LOO strategy is therefore better than the subregion strategy if we evaluate the efficiency in terms of the similarity of the RPP estimates.

The validation efficiency, quality, and reliability of the REVEALS estimates are shown by the SEs of the RVs. There are two sources of uncertainty in the RVs: the SDs of RPP values and the between multiple site variation in pollen assemblages. Both are incorporated into the SEs of the RVs. Our results show that the SEs of RVs for all taxa are lower than RV values. The coefficients of variation between observed and estimated vegetation abundance (expressed as standard variation divided by the OBV values) vary among taxa. The largest variation detected between RVs and OBVs is less than 1.4-fold, whereas it is as large as 8.19-fold between PPs and OBVs in strategy I. Similar trends are found in strategy II, where the greatest difference between RVs and OBVs is less than 1.1-fold, whereas it is 7.1-fold between PPs and OBVs. In general, the RV of all taxa, except *Pinus* in both strategies, show better results than PP alone, which confirms that the application of RPP estimates in the REVEALS model provides consistently better and more reliable estimates of vegetation cover. Overall, the tests confirm the reliability of the RPP estimates, and in the case of this study, the LOO strategy is the best choice for evaluating the RPP estimates using the REVEALS model.

This study has shown that herb taxa, and in particular *Artemisia*, Caryophyllaceae, and Poaceae, are overrepresented by the RVs. Some herbs match well (Asteraceae S.F Cichorioideae and *Humulus*). Tree taxa (*Pinus*, *Quercus*, *Robinia*, and *Ulmus*), although not *Castanea*, are underrepresented by the RVs. This is because pollen records from small-sized sites capture more pollen grains from plants in close proximity than those from farther away (Prentice, 1985; Sugita, 1994), and additional trees growing at a longer distance are underestimated in the mean regional estimated vegetation by the REVEALS model. Similar trends are detected in the mountain region of the northern Pyrenees (Marquer et al., 2020). In addition, the overrepresentation of herbs and underrepresentation of trees by the REVEALS model shows larger dissimilarity for aggregated taxa (herbs vs. trees) than individual taxon (e.g., Marquer et al., 2014).

Pollen assemblages represent the surrounding plant abundance well in terms of individual taxon although the degree of similarity between observed and REVEALS-estimated vegetation abundance varies among taxa. The highest similarity between OBVs and RVs is found in taxa with high abundances in both pollen and vegetation, e.g., Poaceae, *Artemisia*, and Caryophyllaceae. The discrepancies between RVs and OBVs are much higher for taxa with a low abundance of pollen or vegetation, such as *Humulus*. In general, the RVs improve the accuracy of quantitative reconstruction of vegetation around the sampling sites in comparison with PPs, which supports earlier validation studies in China (e.g., Wan et al., 2022) and Europe (e.g., Hellman et al., 2008a; Hellman et al., 2008b), although further tests using pollen records from large lakes are necessary.

We attempted a randomized splitting of the sites to validate our RPPs, but obtained high SDs in our RPP estimates and large SEs in the REVEALS results, most likely owing to the heterogeneity in the study region of local vegetation. We therefore only used dataset splitting by region.

#### 4.2.2 Spatial representation of the REVEALS model

Our study revisits one of the questions in the quantitative reconstruction of regional vegetation, the spatial representation of REVEALS based vegetation abundance. A number of empirical studies have been conducted in Europe (Soepboer et al., 2007; Hellman et al., 2008b; Hjelle et al., 2015; Marquer et al., 2020) and China (Wan et al., 2022) to address this issue, and the spatial representation was estimated to vary between 25 and 200 km distance from the sampling basin. The RVs based on pollen records from small- to middle-sized lakes in the Swiss plateau are comparable with vegetation collected around 200 km (Soepboer et al., 2007). The spatial extent of the RVs using pollen records from large lakes is around 50 km x 50 km to 100 km x 100 km (ca. 25-50 km radius), although the best fit between RV vegetation and observed vegetation within the 100 x 100 km area in southern Sweden (Hellman et al., 2008b), while RVs from small water reservoirs in tropical China show the highest similarity with vegetation collected within 80 km, although there are no significant differences between observed vegetation collected within 50 km and 80 km (Wan et al., 2022).

The spatial size of the vegetation dataset required for validation of REVEALS estimates of plant cover and/or RPPs is an issue that was first evaluated and discussed by Hellman et al. (2008b) for pollen records from large lakes. No comparable study has been performed since in other regions. It has been assumed that the spatial scale of REVEALS reconstructions is of similar magnitude independently of the characteristics of the regional vegetation (e.g., Trondman et al., 2015; Githumbi et al., 2022). In theory and practice, however, the strict definition of the pollen source area is difficult for REVEALS application. Sugita (2007a) defined it as the area within which most of the pollen comes from. The usage of multiple small moss polster sites for estimating regional vegetation is the most notable difference between this study and previous work. The high homogeneity of vegetation collected from the vegetation map in the sampling region induced high similarity in the mean observed regional vegetation between different distances although the actual landscape is much patchy. Nevertheless, our results demonstrate that herb covers estimated by REVEALS are more similar to the mean observed vegetation within 75 km, whereas RVs of tree taxa, except *Robinia*, are closer to the mean vegetation within 100 km in strategy I. Notably, the similarity between RVs and OBVs collected from different distances varies from individual taxa in strategy II, RVs of *Artemisia*, and Asteraceae SF. Cichorioideae are much more similar to OBVs within 100 km, whereas RVs of *Humulus* and *Ulmus* are more similar to OBVs within 125 km, and RVs of *Pinus*, Poaceae, *Quercus*, and *Robinia* are closer to OBVs within 150 km. Overall, the RVs of herb taxa are more similar to OBVs collected within shorter distances, whereas the RVs of trees are more similar to OBVs collected within longer distances in both strategies. Our findings point to the importance of collecting vegetation from different distances in evaluating the REVEALS-estimated regional vegetation and that the spatial extent of the REVEALS model is related to the character of the taxa and the size of the “deposition basin”.

#### 4.2.3 Discrepancies between RVs and OBVs

The REVEALS model results in much better estimates of vegetation cover than unconverted pollen percentages; however, discrepancies still exist between estimated and observed vegetation. This is most clear for taxa that are dominant in some sites but absent in the others, such as *Robinia* and *Castanea*, whose RPP values obtained in the testing process may not represent the correct average value for this region. The RVs slightly overestimate most of the herb taxa and underestimate all tree taxa in strategy I, and RVs underestimate *Pinus*, *Robinia*, and Asteraceae SF. Cichorioideae, whereas they overestimate Poaceae, Caryophyllaceae, and *Ulmus* in strategy II.

Sugita (2007a) suggests that the number and properties of sites that are necessary for REVEALS to reconstruct representative regional vegetation depend on the regional spatial complexity, gradients, species composition and diversity, and basin size. A variety of factors could account for the discrepancies between observed and estimated vegetation from the REVEALS model, and we consider the most important ones to be (1) pollen assemblages; (2) between-site variation in the vegetation of multiple sites; and (3) other factors related to methodology.

##### 4.2.3.1 Pollen assemblages

The temporal differences in the pollen sample and the collected vegetation might be responsible for some of the discrepancies between the estimated and observed vegetation. Moss polsters represent generally 1–2 (3) years of pollen deposition according to studies in Europe (e.g., Cundill, 1991; Räsänen et al., 2004). Given the small green fresh moss samples we collected in Shandong, we believe that the moss polsters represent vegetation from no more than 2 years. However, the time span of the obtained vegetation abundance varies, where the local vegetation applied in the ERV model is based on 1 year, whereas the regional vegetation is collected from the available vegetation inventories conducted in the 1980s (Hou, 2019). Nevertheless, there is little evidence to believe that the discrepancies between observed and estimated vegetation are due to this factor, and the application of regional vegetation from the available vegetation mapping does not necessarily affect the validation.

The second aspect is the difference in the gradients of the target taxa in pollen and vegetation. The validation is applied on a few taxa with considerable amounts both in pollen and in vegetation (accounting for 67%–99% of the total pollen assemblages). However, the dominant taxa vary in plant abundance among our sites, and several taxa (e.g., *Vitex, Ziziphus*, and *Zanthoxylum bungeanum*) have significant cover in the vegetation survey but are rarely present in pollen assemblages. These taxa are therefore not selected for validation. The variation between sites with larger proportions of unselected taxa enhances between-site variation and in turn results in larger SEs of the REVEALS estimates. However, all RVs obtained are with relatively low SEs, and none of the SEs are larger than the RVs in both strategies. Therefore, it is difficult to test if the absence of unselected taxa is responsible for the discrepancies between observed and simulated vegetation abundance.

##### 4.2.3.2 Vegetation data

Another important and probably more crucial issue is the precision of the estimates of plant abundance, which varies depending on the source and temporal resolution of the vegetation data. One of the caveats of this study is the use of a low spatial resolution vegetation map. To reduce the SE of the RVs, multiple small sites (in strategy I) and the mean RVs of the 36 REVEALS runs based on a single small site (strategy II) were used to estimate regional vegetation. Mean OBV collected from vegetation maps was used to compare with RVs. However, empirical vegetation survey data in anthropogenic landscapes might not always fulfill the requirements of the ERV and the REVEALS model, such as the important condition of stationarity of the spatial structure of vegetation and plant communities of study areas locally and regionally within the study region at regional spatial scale, although the coarse resolution of the regional vegetation cannot be effectively attributed to spatial variation in vegetation composition at finer scales.

Our results show that the RV has a very good fit with OBV mainly for *Pinus, Artemisia*, Poaceae, *Robinia*, and Asteraceae SF Cichorioideae; the similarity between estimated and observed vegetation is less good for some other taxa, and cultivation may be responsible for some of the discrepancies. The cover of categorized communities, extracted from available vegetation inventories conducted in the 1980s (Hou, 2019), may have changed, and this is particularly the case for *Castanea*, because *Castanea* preferably grows at the foot of the mountains at low altitudes. The abundance *of Castanea* cultivation has probably increased over the last 40 years due to the extension of farmland through exploring new territory at the foot of the mountains in the study region. Therefore, the validation of RPP estimates needs to be applied to an adequate temporal resolution and areal extent of vegetation survey in the future.

##### 4.2.3.3 Methodological issues

The Landscape Reconstruction Algorithm, which includes REVEALS as the first step, is developed in ideal circumstances for the sake of simplicity. Highly simplified assumptions include (1) the sedimentary basin for pollen deposition and source plants are located on a flat terrain; (2) there are no spatial gradients in plant distribution and plant community structure in the region, even though vegetation is patchy; (3) wind comes from all directions evenly; (4) pollen grains deposited in the sedimentary basin are all airborne (Sugita, 2007a; Sugita, 2007b). In addition, simulations demonstrate that large basins > 200–500 m in radius are ideal for the REVEALS application for regional vegetation reconstruction (Sugita, 2007a). Our study region is characterized by low mountains, where complex topography limited the availability of large sites, particularly lakes. The application of the REVEALS model on multiple small sites might explain some of the uncertainties of the estimated vegetation abundance in the mountain region as discussed above.

There are strengths and weaknesses in both strategies that have been used here. One of the potential drawbacks of strategy I of reducing the number of sites for the ERV model is that it can change the occurrence frequency of target taxa, resulting in shorter gradients. It is not possible to produce reliable RPP estimates for target taxa if there are poor correlations between pollen and vegetation, although there may be strong gradients within all sites in the region to build up a strong pollen–vegetation relationship.

Understanding and quantifying the representation of vegetation by pollen was the focus in the development of the ERV model, and also one of the major goals of pollen analysis. Despite the issues discussed above, the RPP values for nine of the 10 target taxa are validated as reliable approximations in terms of pollen representation of vegetation.

### 4.3 Comparison with other RPP validations in China

Three studies have compared and evaluated RPP estimates in China (Xu et al., 2014; Jiang et al., 2020; Wan et al., 2022). Regardless of RPP selections, all studies found that vegetation cover estimates using either REVEALS alone or both the REVEALS and LOVE models provide a better representation of vegetation than pollen proportions.

Of the three studies, the one conducted in the steppe region in Inner Mongolia found that predicted and observed vegetation abundances match well for most of the pollen taxa evaluated (12 among 18 taxa) and that the RPP estimates for those taxa are regarded as reasonable (Xu et al., 2014), although the major caveat of their validation was the circular use of the datasets, without considering spatial autocorrelation. The high similarity between the RPP estimates in our study and those of Xu et al. (2014) raises the possibility for future validation using the pollen records and observed regional vegetation from one study in combination with RPP estimates obtained from the second study. 

The RPP estimate validation study in subtropical China of Jiang et al. (2020) found that both local and regional vegetations are closer to observed vegetation than the pollen proportions alone. They estimated regional mean vegetation abundance by the application of the REVEALS model on pollen records from multiple small sites, and the local plant abundances by the application of the LRA with the leave-one-out cross-validation strategy (Jiang et al., 2020). Similar conclusions were made in tropical China, where the RPP estimates obtained from surface soil were validated by the application of the REVEALS model on extra-surface pollen records from six reservoirs with a radius between 400 and 750 m from the same region to estimate regional vegetation abundance. This study was methodologically sound, as it used an independent dataset for validation (Wan et al., 2022). The strategies that we used within our study (both using LOO, and subdividing samples by region) fulfill the necessary validation requirements of independent dataset validation.

Previous LRA validation studies have shown that the REVEALS model performs better on groups of taxa (i.e., land-cover types) than individual taxa (Hellman et al., 2008a; Marquer et al., 2020), because the discrepancies between REVEALS estimated and observed plant abundance for individual taxa within one group might annul each other within that same group. Our study provides a high level of performance for groups of taxa (e.g., “trees” and “herbs”), but grouping taxa does not provide a better fit between RVs and OBVs than for individual taxa. This is due to the fact that almost all tree taxa are underrepresented in the RVs of this study and herbs are overrepresented, hence grouping by tree or herb taxa aggregates discrepancies rather than obscuring them, unlike previous studies.

## 5 Conclusions

This study evaluated the relative pollen productivities (RPPs) generated from an earlier study of the cultural landscape of Shandong (Li et al., 2017). The results show that the RVs of all taxa are generally closer to OBVs than PPs, and the degree of similarity between RVs and OBVs depends strongly on the taxa composition of the vegetation and pollen assemblages; it is highest for taxa dominant in the region, such as *Artemisia*, Poaceae, and *Humulus*. The RVs of all herb taxa except *Humulus* and Asteraceae SF Cichorioideae are slightly overrepresented, and the RVs of all tree taxa are underrepresented except for *Castanea*. This study confirms the reliability of obtained RPP estimates for nine of 10 taxa, *Artemisia*, *Humulus*, *Castanea*, *Quercus*, Asteraceae SF. Cichorioideae, Caryophyllaceae, *Ulmus*, Poaceae, and *Robinia* for their potential applications in the quantitative reconstruction of vegetation abundance in temperate China.

The comparison between the RVs and OBVs show that the RVs of all herb taxa are more similar to OBVs collected from shorter distances, whereas the RVs of all tree taxa are more similar to OBVs collected from longer distances. This study points to the importance of collecting adequate temporal and spatial resolution vegetation survey data from various distances in evaluating the RVs and that the spatial representation of the REVEALS model is strongly related to the characteristics of taxa, and size of the deposition basin.

This study proposed two new alternatives for RPP estimate validation, which promotes the possibility of evaluating the reliability of the RPP estimates using the REVEALS model. The results show that the LOO strategy is the better approach for evaluating the RPP estimates from surface moss pollsters in this study region. Two better alternatives for evaluating the reliability of the RPP estimates from this data set could be (i) using pollen and vegetation from other sets to evaluate the RPP estimates and (ii) collecting more RPP values to compare with the obtained RPP estimates for the taxa, such as *Castanea*, whose RPP estimates are not reliable from this or other regions. Therefore, comparative analyses of RPP estimates and validation need to be carried out across regions and vegetation types where the RPP estimates are obtained.

## Data availability statement

The original contributions presented in the study are included in the article/**Supplementary Material**. Further inquiries can be directed to the corresponding author.

## Author contributions

FL and M-JG conceptualized and coordinated the study as a contribution to the PAGES working group “LandCover6k” and collected the pollen samples and vegetation survey in the field. FL, M-JG, and SS designed the validation strategies, SS solved all specific issues related to the application of the ERV and REVEALS model. FL prepared pollen samples, counted pollen, and had the major responsibility of vegetation data collection and handling. FL prepared the first draft of the manuscript and all figures and tables and finalized the manuscript for submission. RF and SS edited the manuscript. All coauthors contributed comments about and corrections to the manuscript. All authors contributed to the article and approved the submitted version.
