# Supplementary material for: Evaluation of relative pollen productivities in temperate China for reliable pollen-based quantitative reconstructions of Holocene plant cover
Source: Front Plant Sci. 2023 Nov 7;14:1240485. doi: 10.3389/fpls.2023.1240485 (PMC10662289; doi:10.3389/fpls.2023.1240485)
Supplement: Supplementary file 1 [file Image_1.pdf]

Validation of relative pollen productivities for plant indicators of past human-induced vegetation in temperate China for reliable pollen-based quantitative reconstructions of Holocene plant cover

Furong Li[1,2], Marie-José Gaillard[2], Siqi Xie[1], Kangyou Huang[3], Qiaoyu Cui[4], Ralph Fyfe[5], Laurent Marquer[6], Shinya Sugita[7]

Electronic supplementary material

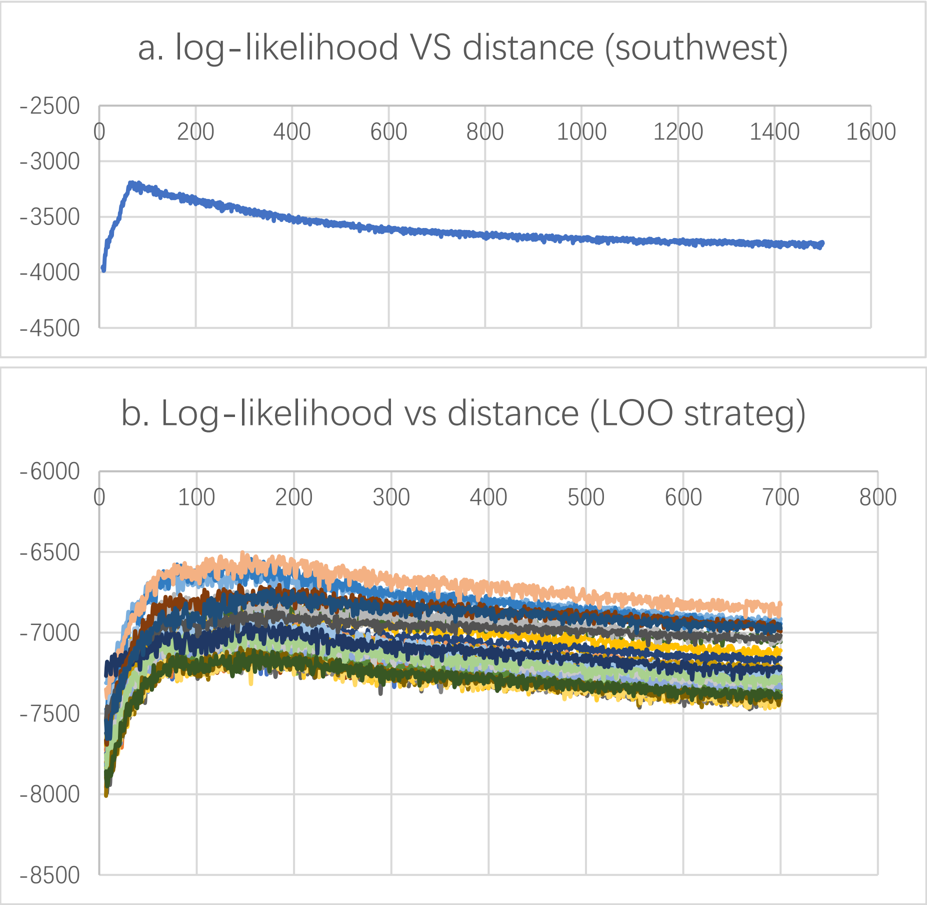

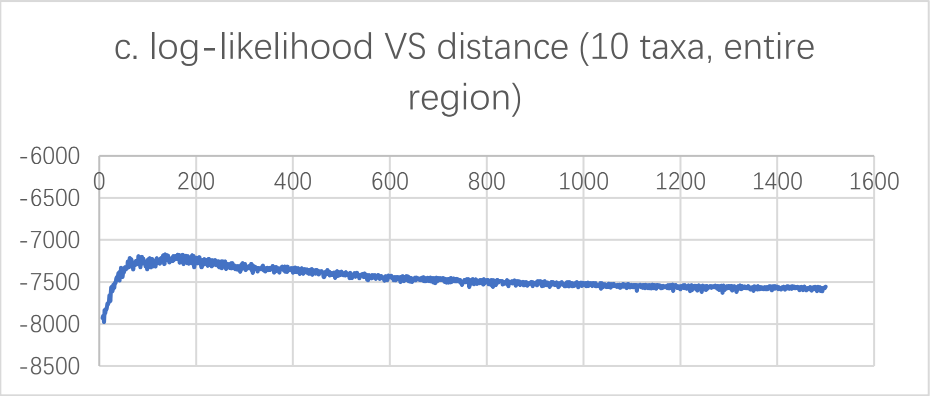

Figure S1. Plots of the log-likelihood against distance in the ERV runs in combination of ERV sub-model 3 with Prentice's taxon-specific distance weighting. The upper, middle, and lower panels represent results based on pollen and vegetation data in strategy I (a), strategy II (LOO; b), and the entire region (c).
